# Comparative Performance Analysis of Five Magnetometers for Geomagnetic Monitoring

**DOI:** 10.3390/s26154700

**Published:** 2026-07-23

**Authors:** Guillermo Caro, Enrique Carrasco, Joaquín Diaz Peña, Benjamín Urra, Enrique Rojo, Loreto Castro, Manuel Bravo, Victor Pinto, Marina Stepanova, Adán Godoy, Elías Ovalle, Clezio Marcos Denardini, Sony Su Chen

**Affiliations:** 1 Centro de Instrumentación Científica, Universidad Adventista de Chile (UNACH), Chillán 3780000, Chile; guillermo.caro@unach.cl (G.C.); benjaminurra@unach.cl (B.U.); enriquerojo@unach.cl (E.R.); manuelbravo@unach.cl (M.B.); adan.godoy@unach.cl (A.G.); eliasovalle@unach.cl (E.O.); 2 Space and Planetary Exploration Laboratory, Universidad de Chile (UCHILE), Santiago 8370451, Chile; joaquindiaz@uchile.cl; 3 Departamento de Matemática y Física, Liceo Bicentenario de Excelencia Polivalente San Nicolás, San Nicolás 4020000, Chile; loretocastro@liceosannicolas.cl; 4 Physics Department, Universidad de Santiago de Chile (USACH), Santiago 9170124, Chile; victor.pinto@usach.cl (V.P.); marina.stepanova@usach.cl (M.S.); 5 Instituto Nacional de Pesquisas Espaciais (INPE), São José dos Campos 12227-010, SP, Brazil; clezio.denardin@inpe.br (C.M.D.); sony.chen@inpe.br (S.S.C.)

**Keywords:** earth’s magnetic field, fluxgate magnetometer, magneto-inductive magnetometer, geomagnetic monitoring, low-cost sensors, magnetometer comparison, noise analysis, signal stability, temperature effects

## Abstract

Magnetometers are essential for monitoring the Earth’s magnetic field in applications such as geophysics, space weather, navigation, and volcanic studies. While professional-grade instruments provide high reliability, their cost limits spatial coverage, particularly in remote regions. This study presents a comparative analysis of five ground-based magnetometers deployed simultaneously at Universidad Adventista de Chile (36.64° S, 71.99° W) from 5–8 August 2025. Noise, signal stability, and response to geomagnetic variations were evaluated, emphasizing the influence of ambient temperature. Fluxgate and subsurface-deployed instruments showed higher consistency and reduced thermal sensitivity, while low-cost magneto-inductive sensors exhibited strong linear temperature dependence, sometimes with temporal delays. Inter-instrument correlation analyses highlighted performance differences and the trade-offs between cost and reliability. The results demonstrate that low-cost magnetometers, if carefully deployed and calibrated, can complement professional-grade sensors, improve spatial coverage, and provide valuable geomagnetic observations in regions with limited infrastructure and logistical constraints.

## 1. Introduction

Magnetometers are essential instruments for measuring variations in the Earth’s magnetic field and are widely used in geophysics, space weather monitoring, navigation, and satellite orbit propagation, besides their relevance for environmental and volcanic studies [1,2]. Ground-based magnetometer observations are critical for characterizing geomagnetic activity, including magnetic storms, substorms, and quiet-time variations, as well as for long-term monitoring of regional and secular magnetic field changes [3,4,5].

A wide range of magnetometer technologies are currently in use, including fluxgate, Overhauser, proton precession, and optically pumped sensors [6]. These instruments differ in sensitivity, noise characteristics, temporal stability, dynamic range, and susceptibility to environmental effects, leading to different responses when observing the same geomagnetic signal. Consequently, quantitative intercomparisons under common measurement conditions are necessary to assess instrumental performance and ensure data consistency across different sensors and networks. Although magnetometer networks are widely deployed worldwide, direct performance comparisons of different instruments remain relatively limited. Such studies are particularly important for applications that rely on subtle magnetic field variations, including space weather monitoring, long-term trend analysis, volcanic monitoring and magnetotellurics, where instrumental biases and noise levels can significantly impact data interpretation [7,8].

Magnetometers for scientific purpose, with high-quality core sensors and steep hysteresis curve are capable of adequately mitigating environmental effects, particularly temperature-induced fluctuations through the use of external components and controlled installations. Such classes of instruments have an estimated cost starting at approximately USD 20,000 [9]. As a result, these systems remain inaccessible for many universities and research centers, leading to fewer operational stations and reduced spatial coverage. This limitation is especially pronounced in the Southern Hemisphere, where rugged terrain and the remoteness of available land at high southern latitudes, such as southern continental Chile and Antarctica, impose additional technical and logistical challenges for the deployment and maintenance of geomagnetic observatories.

In the South American sector, several geomagnetic stations have been deployed to monitor magnetic field variability under the influence of both global and regional effects.

In Chile, geomagnetic stations are supported by several regional and international initiatives. The South American Meridional B-field Array (SAMBA) [10] has provided a meridional chain of magnetometers along the Chilean sector, enabling latitudinal studies of geomagnetic field variability. In addition, a geomagnetic observatory on Easter Island is part of the International Real-Time Magnetic Observatory Network (INTERMAGNET) [11] and provides high-quality reference measurements in the southeastern Pacific sector. Furthermore, the EMBRACE Magnetometer Network (Embrace MagNet) [12] operates a magnetometer in Chillán, contributing complementary regional observations in South America. In particular, the EMBRACE magnetometer installed in Chillán, Chile, has been used in recent studies to characterize magnetic fluctuations during quiet geomagnetic conditions and solar minimum periods, highlighting its relevance for regional space weather monitoring [13]. Magnetometers are also employed in Chile for volcanic monitoring, supporting the detection of magnetic field variations associated with volcanic processes in a tectonically active region [14].

However, several SAMBA stations are currently no longer operational due to the expiration of their original funding, highlighting the need for more sustainable geomagnetic monitoring solutions. In this context, the evaluation of new-generation and low-cost magnetometers has become increasingly relevant, as such instruments may enable denser spatial coverage and improved long-term continuity of observations. Chile’s pronounced North–South geographic extent further makes the country particularly well suited for latitudinal studies of geomagnetic field variability during solar-driven events.

In this work, we present a comparative performance analysis of five ground-based magnetometers operated under similar observational conditions. The comparison focuses on key instrumental metrics relevant for geomagnetic monitoring, including noise characteristics, signal stability, and response to geomagnetic variability, to assess their suitability for long-term and event-based applications.

## 2. Instrumentation and Experimental Setup

Five different types of ground-based magnetometers were co-located and operated simultaneously during a short-term measurement campaign conducted on the campus of the Universidad Adventista de Chile, located in Chillán, Ñuble Region (36.64° S, 71.99° W), between the 5th and the 8th of August 2025. This measurement campaign was carried out in the framework of the III Workshop on Scientific Instrumentation in Geosciences, which provided an appropriate setting for coordinated instrument deployment and intercomparison activities. The instruments were installed within proximity to each other (3 to 5 m), ensuring that all sensors were exposed to nearly identical geomagnetic and meteorological conditions. Any residual coupling due to their proximity is expected to affect only the high-frequency components of the measurements and should not have a significant impact on the observed diurnal magnetic field variations. This co-location strategy minimizes spatial magnetic field gradients and allows observed differences in the recorded signals to be attributed primarily to instrumental characteristics rather than to geophysical variability.

The measurement campaign was designed to compare magnetometers representing a range of sensor technologies and operational concepts currently used or considered for geomagnetic monitoring applications. Operating all instruments at the same site and time interval enables a direct and objective comparison of their performance under real geomagnetic conditions representative of quiet-time geomagnetic variability, rather than being influenced by major geomagnetic storms or extreme disturbances.

Given that some of the magnetometers were installed at the surface, above ground, and others buried (according to instrument design), ambient temperature variations were monitored as part of the measurement campaign. Temperature measurements were provided by an in situ meteorological station (model PCE-FWS 20N from PCE Instruments) deployed at the same site. These data were used to assess the potential influence of thermal fluctuations on sensor offsets, sensitivity, and noise characteristics, particularly for instruments more exposed to environmental conditions. Table 1 summarizes the meteorological and geomagnetic conditions recorded during the event. The campaign was characterized by stable weather with no precipitation, with ambient temperatures ranging from a nighttime minimum of 0.9 °C to a daytime maximum of 20.4 °C. Geomagnetic activity remained relatively low during the period, reaching a minimum Dst index = −38 nT and a maximum ap index = 48 toward the end of the measurement window, indicating the occurrence of a moderate geomagnetic disturbance during the final hours of the campaign [15].

Table 2 summarizes the main characteristics of the five magnetometers considered in this measurement campaign, including sensor type, institutional ownership, measurement components, sampling rate, and installation conditions. This overview provides the contextual framework for the detailed analysis presented in subsequent sections.

**Figure 1:**
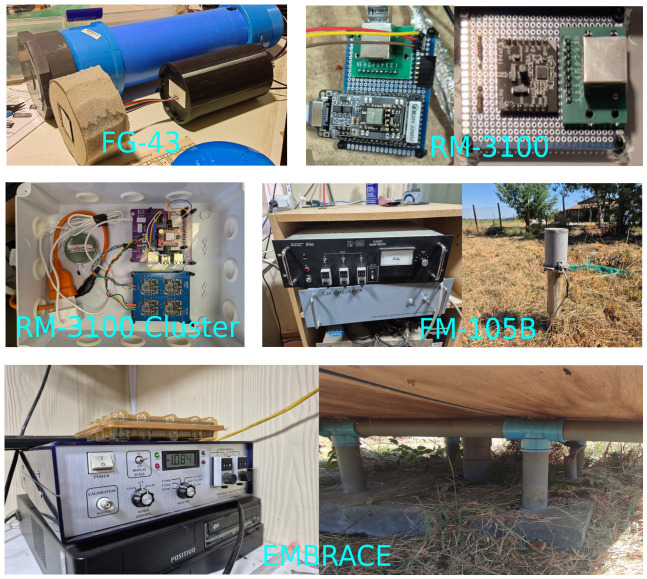
Pictures of the magnetometers (**top**) FG-43 and RM3100, (**center**) RM3100 Cluster and FM-105B, (**bottom**) EMBRACE.

The following subsections briefly describe each magnetometer showed in Figure 1, focusing on its measurement principle, installation characteristics, and relevant technical specifications, whereas wiring diagrams of each magnetometer are shown in Supplementary Material. These descriptions are intended to support the interpretation of the comparative results discussed later in the paper. It is important to note that the altitude differences reported in Table 2 are small compared with the spatial scale of the geomagnetic field variations being measured and, therefore, are not expected to have any significant effect on the observed variations of the Earth’s magnetic field. Moreover, magnetometers Section 2.1, Section 2.2 and Section 2.3 were factory calibrated, whereas Section 2.4 and Section 2.5 were not calibrated before deployment. This aspect is intentionally retained in the analysis to evaluate the performance of low-cost and minimally calibrated sensors under realistic field conditions.

### 2.1. EMBRACE Fluxgate Magnetometer

Geomagnetic measurements were conducted using a three-axis fluxgate magnetometer from the EMBRACE MagNet, operated by the National Institute for Space Research (INPE). This network monitors geomagnetic field variations across South America, focusing on space weather phenomena and regional magnetic disturbances. The specific unit employed has been operating in Chillán (36.64° S, 71.99° W) since 2019, installed underground to mitigate thermal fluctuations and surface-level environmental noise.

This magnetometer was developed at the Jicamarca Radio Observatory (JRO). The instrument measures vector components of the geomagnetic field using three orthogonal fluxgate sensors, with typical operational parameters including a total range up to ±75,000 nT, multiple dynamic ranges (e.g., ±250 nT to ±2500 nT), a sensitivity of approximately 2.5 mV/nT, and a resolution of 0.1 nT [12]. Such characteristics make it suitable for monitoring both quiet and disturbed geomagnetic conditions. For data acquisition, the instrument provides a digital output interface connected to a PC running proprietary software. Raw measurements are internally acquired at a sampling rate of 1 Hz; however, calibrated magnetic field values expressed in nT are output and archived at an effective sampling rate of one of one sample per second, being averaged to achieve 1 min resolution afterwards.

### 2.2. FM-105B Fluxgate Magnetometer

This is a type of magnetometer developed by EDA Instruments Inc. around 1980 in Canada. The model used is the FM105-B. It has a compensation range of 0 to ±40,000 nT for X and Y axes and 0 to ±70,000 nT for Z axis, all components in two ranges with a switch selectable and resolution of 100 nT/Volt [16]. The control unit incorporates three precision potentiometers to compensate for the local magnetic fields manually, with a locking mechanism and counter. The temperature compensation is made automatically by a thermistor on the head unit with 100 ohm and 3900 ppm/C. The fluxgate element has an excitation voltage of 0.25 V with a frequency of 400 Hz, with a range of 150 to 1000 Hz. The output analog voltage is recorded on a microcontroller with RTC, microSD module, and ADC board by Mayhew Labs equipped with LTC185x. The microcontroller is programmed to save data of each component at a rate of 1 Hz in a simple 4-column ASCII text file format (YYYY-MM-DD-HH-MM-SS X Y Z).

### 2.3. FG-43 Fluxgate Magnetometer

The third instrument deployed during the measurement campaign was a commercial three-axis fluxgate magnetometer, model FG-43, manufactured by FG Sensors (Slovenia). According to the manufacturer’s datasheet, the FG-43 system provides vector measurements of the geomagnetic field with a typical sensitivity of approximately 0.1 nT per axis and a measurement range of ±80,000 nT per axis. Data acquisition can be performed at sample rates up to 33 Hz, and the instrument includes integrated signal conditioning and a data logger with USB/SD card interfaces [17]. The FG-43 also incorporates internal temperature monitoring over an extended range, supporting stable operation in field environments.

During the campaign, the FG-43 operated buried underground, alongside the other co-located instruments under identical geomagnetic conditions, providing a commercial benchmark for performance comparison. This benchmarking evaluation serves to assess the feasibility of employing the FG-4 sensor, the core magnetometric element of the FG-43, for the Space Weather School Observatory (OESTE in Spanish) project, which aims to deploy a new instrument incorporating the FG-4 sensor across schools in Chile for space weather monitoring and research [18]. An external logging system was implemented because the burial of the instrument made the task of manually removing the SD card unfeasible. It consisted of a LibreComputer Le Potato connected via serial protocol to the FG-43 through USB, which registered each observation with a UT timestamp in a CSV archive.

### 2.4. RM3100 Magneto-Inductive Magnetometer

The fourth instrument included was the RM3100 magneto-inductive magnetic field sensor, a commercial off-the-shelf device developed by PNI Sensor Corporation. Unlike fluxgate magnetometers, this sensor is based on the magneto-inductance principle, in which variations in the external magnetic field modify the effective permeability of an inductive coil, resulting in changes in its characteristic oscillation time. The circuit consists of an RL oscillator, with the coil parallel to a Schmitt trigger [19].

The RM3100 consists of a module that integrates three orthogonally oriented magneto-inductive circuits, with its coils arranged in a geografically North–East–Down configuration, enabling full vector magnetic field measurements. Signal acquisition and processing are handled by an on-board application-specific integrated circuit of 24-bit for each axis, which can provide digital output to an external microcontroller via I2C or SPI communication protocols. Raw measurements are delivered as digital counts [20].

According to manufacturer specifications and previous works, this sensor offers a measurement range of ±800 µT, low power consumption (<10 mW), and compact physical dimensions (weights < 3 g and has a surface area of 2.54 × 2.54 cm^2^), making it suitable for low-cost geomagnetic applications. Its sensitivity, noise level, and maximum sampling rate can be configured through an internal integration parameter known as cycle count, achieving values of <13 nT for resolution, <15 nT for noise, and >500 Hz for sampling rate [21]. For this campaign, the sensor was housed in a waterproof box, connected to an ESP32-S3 development board via I2C, capturing the data corresponding to the average of the measurements for one second. This information was sent in real time via serial communication to a Raspberry Pi 4 model B computer and stored as a text file.

### 2.5. RM3100 Cluster Magnetometer

In addition to the single RM3100 unit described above, a multi-sensor RM3100 cluster magnetometer was deployed during the campaign. The cluster consisted of eight RM3100 sensors arranged at the vertices of a rectangular prism with a square base and a shorter vertical dimension. The approximate center-to-center spacing was 3.5 cm along the base edges and 3.0 cm along the vertical direction. All sensors were oriented consistently so that the vector measurements could be combined directly [22]. Each RM3100 equipment samples at 37 Hz. A Raspberry Pi 5 served as the central acquisition computer, with timing referenced to a JAVAD TRH G2P GPS receiver [23] using NMEA time messages.

The sensors were mounted on a custom PCB assembly designed so that each RM3100 was positioned directly above an MCP9808 [24] digital temperature sensor. The full assembly was housed in a sealed, weatherproof enclosure to mitigate humidity exposure. During the campaign, the enclosure was deployed at the surface (not buried). Data was logged continuously in CSV format, with one file per magnetometer. Each record contained a Unix timestamp, three-axis magnetic field measurements in raw digital counts, and the temperature (degrees Celsius) measured by the MCP9808 co-located with that RM3100 sensor.

The data had to be extensively cleaned due to a software error that altered the axis of measurements of individual magnetometers. It has not been possible to replicate the error upon further laboratory experiments and additional operations have shown no errors. The data was cleaned by analyzing the error spectrum and eliminating data on a per axis basis, as the software error manifested itself in increased high-frequency residuals that allowed for defining areas of bad data.

Although the cluster architecture was conceived to support multichannel interference mitigation methods (for example PCA- or ICA-based approaches [25]), the analysis in this work uses a simple average across the eight RM3100 sensors to obtain a representative cluster time series as well as straightforward decimation to get 1Hz data for the final comparison.

## 3. Results and Discussion

The data analysis focuses on evaluating the consistency, stability, and temperature sensitivity of the magnetic field measurements recorded by the five co-located magnetometers during the campaign. To enable direct comparison among instruments with different native sampling rates, all data sets were resampled to a common temporal resolution of one value per minute, computed as the median of the original samples within each minute, reducing the influence of outliers while preserving the dominant geomagnetic variability.

**Figure 2:**
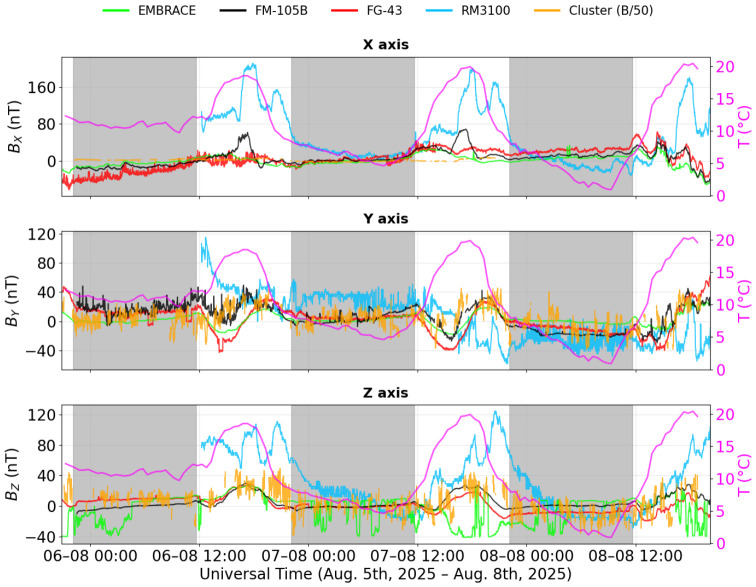
Time series of X, Y, Z magnetic field components from five co-located magnetometers, 5–8 August 2025.

Figure 2 presents the time series of the magnetic field components in geographic coordinates (X, Y, Z) measured by the five instruments over the full duration of the campaign, after removing the corresponding baseline for each magnetometer and component. This normalization removes constant offsets and enables a direct comparison of relative variations among instruments. The baseline values, summarized in Table 3, were computed following the procedure described in [26,27], as the average magnetic field between 23:00 and 02:00 local time. Table 3 also reports the standard deviation and range of the measurements for each instrument and component. The time series are shown at a common temporal resolution of one minute, computed as the median of the original samples (except for the EMBRACE magnetometer).

The ambient temperature measured during the campaign is also displayed in Figure 2 on the right-hand y-axis to support a qualitative assessment of possible thermal effects on the magnetic field measurements. For visualization purposes, the Cluster magnetometer data (orange curve) were divided by a factor of 50 to make their variations comparable with those of the other instruments.

In the $B_{x}$-component, the EMBRACE, FM-105B, and FG-43 magnetometers show similar temporal behavior, capturing the main geomagnetic variations consistently. However, the FM-105B exhibits intermittent overestimations around local midday, which coincide with periods of maximum ambient temperature, as indicated by the magenta temperature curve. This suggests a temperature-related influence on the FM-105B measurements under peak thermal conditions.

The RM3100 magnetometer displays a strong dependence on ambient temperature in the $B_{x}$-component, with variations closely following the temperature evolution. The Cluster magnetometer shows a qualitatively similar behavior, although with smaller amplitude, indicating that it partially reproduces the temperature sensitivity observed in the RM3100.

Similar patterns are observed in the $B_{y}$ and $B_{z}$ components, where most instruments capture comparable relative variations after baseline removal. However, in the $B_{z}$-component, the EMBRACE magnetometer exhibits anomalous behavior characterized by a pronounced diurnal variation that is not present in the FM-105B and FG-43 measurements, unlike what is observed for the other components. This suggests a component-specific instrumental issue affecting the vertical field measurement.

Additionally, the temperature dependence observed in the RM3100 appears to include a temporal delay relative to the ambient temperature variations. This phase lag is evident in Figure 2, suggesting that the sensor response is influenced by cumulative thermal effects or thermal inertia associated with the instrument or its installation, rather than by instantaneous temperature changes alone.

Table 3 shows clear differences in both the magnitude and variability of the magnetic field measurements among the instruments. In particular, the RM3100 and RM3100 Cluster exhibit significantly larger standard deviations and ranges compared to the fluxgate magnetometers, indicating higher variability and increased sensitivity to environmental conditions. In contrast, the EMBRACE and FG-43 sensors display lower dispersion values, reflecting more stable measurements under identical conditions.

**Figure 3:**
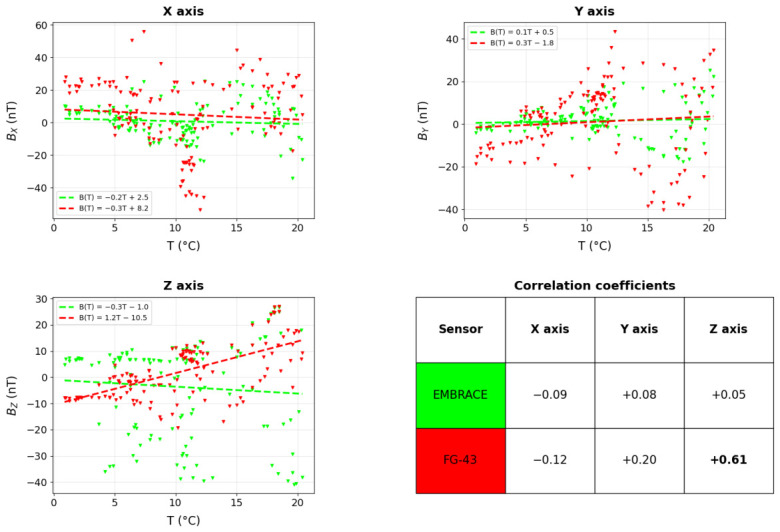
X, Y, Z magnetic field components after median removal, with ambient temperature. Bold coefficients indicate the maximum correlation value for each axis.

Figure 3 presents an analysis of the relationship between the magnetic field measurements and the ambient temperature for the buried magnetometers (EMBRACE and FG-43). Scatter plots are shown for each magnetic field component ($B_{x}$, $B_{y}$, $B_{z}$) as a function of temperature, together with linear regression fits that provide a first-order estimate of temperature dependence. The accompanying table reports the Spearman correlation coefficients, providing a robust measure of monotonic relationships. Note that the high correlation of the Z component of FG-43 is due to the fact that this component is usually similar to the diurnal variation of the temperature.

The results show weak correlations between magnetic field variations and temperature for both instruments, indicating that subsurface deployment effectively reduces thermally induced effects and enhances measurement stability.

**Figure 4:**
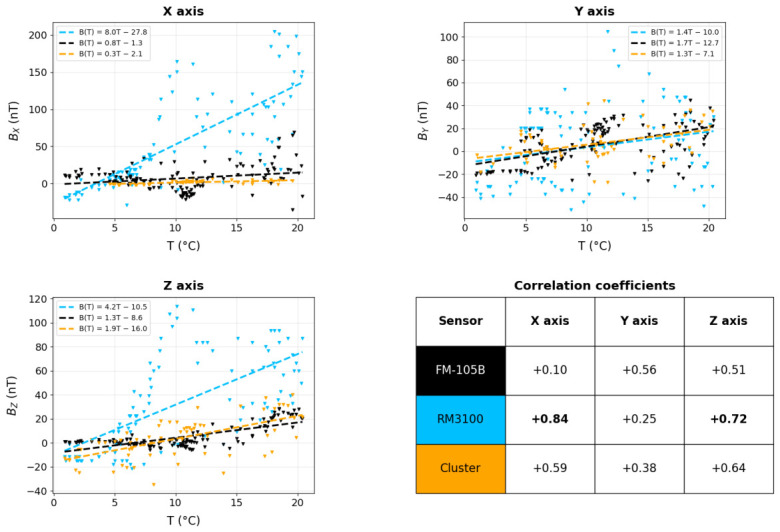
Magnetic field vs. temperature for each component ($B_{x}$, $B_{y}$, $B_{z}$) with linear fits and Spearman correlations. Bold coefficients indicate the maximum correlation value for each axis.

Figure 4 presents the same analysis for the surface-installed magnetometers (FM-105B, RM3100, and RM3100 Cluster). In contrast to the buried sensors, these instruments exhibit a stronger linear dependence on temperature, particularly in the $B_{x}$ and $B_{z}$-components of the RM3100. The Cluster magnetometer shows a similar, though more variable, behavior.

This clear separation between buried and surface-installed instruments highlights the strong influence of installation conditions on temperature sensitivity, confirming that sensors deployed at or near the surface are significantly more affected by ambient thermal variations.

**Figure 5:**
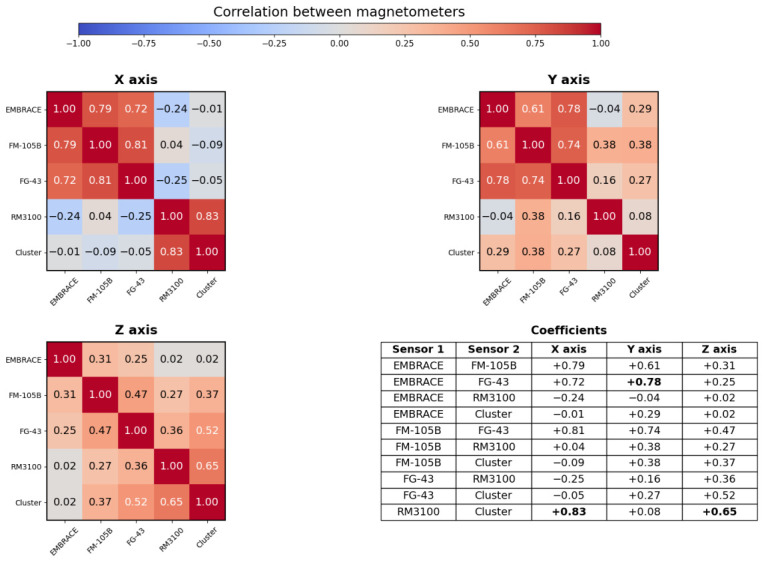
Spearman correlation coefficients between each magnetometer for X, Y, Z components. Bold coefficients indicate the maximum correlation value for each axis.

Figure 5 presents the Spearman correlation coefficients computed between each pair of magnetometers for all three magnetic field components ($B_{x}$, $B_{y}$, $B_{z}$). This inter-instrument analysis provides a quantitative measure of consistency when observing the same geomagnetic signal under identical environmental conditions. Higher correlation values indicate similar temporal responses and effective tracking of common geomagnetic variations, whereas lower correlations reflect the influence of instrumental noise, temperature sensitivity, installation conditions, or calibration differences.

Figure 5 reveals distinct correlation patterns depending on the magnetic field component. For the $B_{x}$-component, the EMBRACE, FM-105B, and FG-43 magnetometers exhibit strong agreement (r > 0.7), indicating a consistent response to the common geomagnetic signal. A strong correlation is also observed between the RM3100 and the RM3100 Cluster, as expected given their shared magneto-inductive sensing technology.

In the $B_{y}$-component, strong agreement is observed between the EMBRACE and FG-43 magnetometers. The FM-105B exhibits weak correlation with EMBRACE, but a comparatively stronger correlation with the FG-43, consistent with its previously identified temperature sensitivity.

For the $B_{z}$-component, the correlation structure changes notably: the FM-105B and FG-43 exhibit weak mutual correlation, whereas the EMBRACE magnetometer shows reduced agreement with the other instruments. This result is consistent with the anomalous behavior observed in the EMBRACE $B_{z}$-component in Figure 2, suggesting a component-specific instrumental issue. Additionally, the $B_{z}$-component of the RM3100 shows relatively high correlation with the FM-105B, which may be attributed to their shared sensitivity to ambient temperature variations.

These results, together with the temperature-dependent behavior identified in Figure 3 and Figure 4, highlight the critical role of thermal effects in the performance of ground-based magnetometers, particularly for sensors deployed at or near the surface. The presence of clear linear temperature dependencies in several components, quantified by the regression slopes (in nT/°C) as a measure of thermal sensitivity and summarized in Table 4, indicates that environmental conditions can introduce systematic variations that may obscure subtle geomagnetic signals. Consequently, installation strategy and thermal control emerge as key factors when evaluating the suitability of different magnetometer technologies for geomagnetic monitoring and space weather applications.

To further quantify the impact of installation conditions, the mean thermal sensitivity was computed for each group. Buried magnetometers exhibit consistently low values, with average absolute slopes of approximately 0.25, 0.20, and 0.75 nT/°C for the $B_{x}$, $B_{y}$, and $B_{z}$ components, respectively. In contrast, surface-installed instruments show substantially higher sensitivities, with mean values of 3.03, 1.47, and 2.47 nT/°C for the same components. This corresponds to an increase of approximately 12, 7, and 3 times, respectively, highlighting a strong amplification of temperature-induced effects in surface deployments. This result quantitatively confirms the effectiveness of subsurface installation in mitigating thermal contamination in geomagnetic measurements.

These results demonstrate that the performance gap between low-cost and professional-grade magnetometers is primarily governed by deployment strategy and thermal stabilization rather than by intrinsic technological limitations. Within the EMBRACE network itself, the Chillán reference fluxgate is buried at 1 m depth inside a non-magnetic enclosure with a sealed underground cable connecting to a remote electronics housing kept under temperature control, a configuration documented to restrict daily temperature differences [12]. When the FG-43 fluxgate sensor (≈ USD$1000) reproduced this subsurface approach during the present campaign, its absolute thermal sensitivities for the horizontal components were 0.3 and 0.3 nT/°C for $B_{x}$ and $B_{y}$, marginally higher than the EMBRACE reference values of 0.2 and 0.1 nT/°C and consistent with the typical 0.1–0.5 nT/°C drift envelope reported for three-axis fluxgate probes used in field operations [28]. For the $B_{z}$ component, the EMBRACE magnetometer exhibited a temporary, component-specific issue during this campaign—manifested as a distorted diurnal variation in $B_{z}$ absent in the FM-105B and FG-43 records—preventing its reliable use as a benchmark. Under this circumstance, comparison with the FM-105B shows that the FG-43 achieved an absolute thermal sensitivity of 1.2 nT/°C, essentially indistinguishable from that of the FM-105B (1.3 nT/°C; Table 4), and a moderate mutual correlation of 0.47, supporting the conclusion that properly installed fluxgate sensors can substantially close the performance gap with professional instruments.

In sharp contrast, the surface-installed RM3100 magneto-inductive sensor exhibited a thermal sensitivity roughly 40 times higher than that of EMBRACE in the $B_{x}$ component (8.0 vs. 0.2 nT/°C; Table 4) and standard deviations 2–5 times larger across all components, with a corresponding 10-fold excess in $B_{x}$ with respect to the FM-105B (8.0 vs. 0.8 nT/°C) and 3-fold excess in $B_{z}$ (4.2 vs. 1.3 nT/°C), although its $B_{y}$ sensitivity (1.4 nT/°C) was actually slightly lower than that of the FM-105B (1.7 nT/°C). Spearman correlations with EMBRACE were close to zero or negative in all components, and correlations with FM-105B were modest ($B_{x}$ = 0.04, $B_{y}$ = 0.38, $B_{z}$ = 0.27; Figure 5), while the high mutual correlation between the RM3100 and the RM3100 Cluster (r = 0.83 in $B_{x}$; Figure 5) confirms a shared sensitivity to ambient temperature rather than a faithful reproduction of geomagnetic variability. A fundamental limitation of the RM3100 is its inability to detect magnetic field variations below the nanotesla level [29]. This observation is consistent with broader characterizations of magneto-inductive technology in low-cost magnetometer networks, where sensors within the same family exhibit high internal consistency but are primarily influenced by thermal bias rather than any genuine enhancement of geomagnetic sensitivity [30]. Low-cost magneto-inductive sensors, therefore, do not yet replicate the performance of professional-grade instruments in their current uncalibrated, surface-deployed configuration, whereas subsurface installation combined with explicit temperature correction enables reliable low-cost fluxgate deployments in regions where the cost of professional equipment is prohibitive.

## 4. Conclusions

This work presented a comparative analysis of five ground-based magnetometers operated simultaneously under identical geomagnetic and meteorological conditions, enabling a direct assessment of their relative performance and temperature sensitivity.

Inter-instrument comparisons reveal clear differences in behavior among sensor types. Fluxgate magnetometers exhibit the highest consistency and correlation across components with coefficients of 0.79, 0.61 and 0.31 between the EMBRACE and the FM-105B; 0.72, 0.78 and 0.25 between the EMBRACE and the FG-43; and 0.81, 0.74 and 0.47 between the FM-105B and the FG-43 for the X, Y and Z axis, respectively. On the other hand, low-cost magneto-inductive sensors show larger variability, although devices based on the same technology display improved mutual agreement, with correlation coefficients of 0.83 in the X axis, 0.08 in the Y axis, and 0.65 in the Z axis between the RM3100 and the RM3100 Cluster.

A key result of this study is the quantification of thermal sensitivity through linear regression slopes. Surface-installed magnetometers exhibit significantly larger temperature coefficients compared to buried ones, with values of 0.10, 0.56 and 0.51 for the FM-105B; 0.84, 0.25 and 0.72 for the RM3100; and 0.59, 0.38 and 0.64 for the RM3100 Cluster for the $B_{x}$, $B_{y}$, and $B_{z}$ components, respectively. In contrast, the buried instruments had correlation coefficients of −0.09, 0.08 and 0.05 for the EMBRACE and −0.12, 0.20 and 0.61 for the FG-43 for the $B_{x}$, $B_{y}$, and $B_{z}$ components, respectively. This confirms that temperature introduces strong systematic effects, particularly in non-buried and low-cost sensors.

Installation conditions are therefore shown to be a primary factor governing measurement stability. Buried sensors consistently exhibit reduced thermal sensitivity and more stable behavior with a linear regression slope of −0.2, 0.1 and −0.3 nT/°C for the EMBRACE and −0.3, 0.3 and 1.2 nT/°C for the FG-43 for the $B_{x}$, $B_{y}$, and $B_{z}$ components, respectively. The magnetometers on the surface showed slopes of 0.8, 1.7 and 1.3 for the FM-105B; 8.0, 1.4 and 4.2 for the RM3100; and 0.3, 1.3 and 1.9 for the RM3100 Cluster for the $B_{x}$, $B_{y}$, and $B_{z}$ components, respectively. Regardless of sensor technology, this demonstrates the effectiveness of subsurface deployment in mitigating environmental contamination.

From a performance perspective, fluxgate magnetometers provide superior stability and robustness, justifying their use in reference-quality observations. In contrast, low-cost sensors are able to reproduce relative geomagnetic variations after appropriate offset removal, offering a favorable price-to-performance ratio for applications requiring dense spatial coverage.

Overall, the results highlight a clear trade-off between cost, stability, and thermal robustness. Fluxgate instruments remain the preferred option for high-precision monitoring, while low-cost magnetometers constitute a valuable complementary solution when combined with appropriate installation strategies and post-processing techniques, particularly in regions with limited infrastructure.

## Figures and Tables

**Table 1:** Meteorological conditions measured in situ and geomagnetic conditions during the campaign; ap index obtained from WDC Kyoto [15].

Parameter	Start	End	Average	Minimum	Maximum
Temperature (°C)	12.3	19.6	10.5	0.9	20.4
Relative Humidity (%)	97.0	58.0	90.2	48.0	99.0
Pressure (hPa)	1019.2	1011.2	1016.9	1011.2	1020.9
ap index	9	48	9.2	2	48

**Table 2:** Characteristics of five magnetometers.

Model	(2.1) EMBRACE	(2.2) FM-105B	(2.3) FG-43	(2.4) RM3100	(2.5) RM3100 Cluster
Institution	INPE	UNACH	UNACH	USACH	UCHILE
Sensor Type	Fluxgate	Fluxgate	Fluxgate	Magneto-inductive	Magneto-inductive
Output/Compose	H, D, Z	X, Y, Z	X, Y, Z	X, Y, Z	X, Y, Z
Start Date and Time (UTC)	August 5th 20:50	August 5th 22:33	August 5th 20:50	August 6th 12:15	August 5th 20:50
Sampling Rate (Hz)	1	1	3	1	37
Initial Value	H: 19,083.1 nT	X: 85.0 nT	X: 19,048.8 nT	X: 21,180.3 nT	X: 1860.3 nT
	D: 3.758°	Y: 7.5 nT	Y: 563.8 nT	Y: 1126.4 nT	Y: 1450.4 nT
	Z: −15,015.5 nT	Z: −45.4 nT	Z: −26,986.1 nT	Z: 13,225.8 nT	Z: 20,488.8 nT
Dynamic Range (μT)	±2.5	±1.0	±80.0	±800.0	±800.0
Resolution (nT)	0.1	0.4	0.4	<13.0	<13.0
Height/Depth (m)	−1.0	+0.5	−1.0	0.0	0.0
Estimated Cost (USD)	25,000	20,000	1000	150	400

**Table 3:** Central tendency and dispersion measures of each magnetometer prior to normalization.

Sensor	Axis	Baseline (nT)	Standard Deviation (nT)	Range (nT)
EMBRACE	X	19,059.7	12.2	85.4
	Y	1237.1	8.3	44.5
	Z	−14,976.0	18.3	68.7
FM-105B	X	98.7	17.7	116.82
	Y	−121.3	16.6	78.8
	Z	2.3	8.9	39.9
FG-43	X	19,095.0	22.4	125.7
	Y	521.5	18.2	105.1
	Z	−26,995.8	9.8	48.70
RM3100	X	21,073.6	59.2	243.9
	Y	1019.9	32.7	172.8
	Z	13,149.2	38.4	152.5
RM3100 Cluster	X	1775.7	122.6	634.7
	Y	−2394.0	755.1	4193.4
	Z	−22,597.0	819.8	4356.0

**Table 4:** Thermal sensitivity of each magnetometer component expressed as linear regression slopes (nT/°C).

Sensor	Installation	$B_{x}$ (nT/°C)	$B_{y}$ (nT/°C)	$B_{z}$ (nT/°C)
EMBRACE	Buried	−0.2	0.1	−0.3
FG-43	Buried	−0.3	0.3	1.2
FM-105B	Surface	0.8	1.7	1.3
RM3100	Surface	8.0	1.4	4.2
Cluster	Surface	0.3	1.3	1.9

## Data Availability

The datasets generated and analyzed during the current study are available at: https://drive.google.com/drive/folders/1vRaOw3E9TMxfwTNobISkrugN_CnllXVu?usp=sharing (accessed on 10 May 2026).

## Supplementary Materials

The following supporting information can be downloaded at: https://www.mdpi.com/article/10.3390/s26154700/s1, Figure S1: Wiring diagram of the FG-43 magnetometer; Figure S2: Wiring diagram of the RM3100 magnetometer; Figure S3: Wiring diagram of the RM3100 cluster; Figure S4: Wiring diagram of the FM-105B magnetometer; Figure S5: Wiring diagram of the EMBRACE magnetometer.

## References

[1] Massey H.S.W., Boyd R.L.F. (1961). The Upper Atmosphere; Reprinted Edition.

[2] Kivelson M.G., Russell C.T. (1995). Introduction to Space Physics.

[3] Gonzalez W.D., Joselyn J.A., Kamide Y., Kroehl H.W., Rostoker G., Tsurutani B.T., Vasyliunas V.M. (1994). What is a geomagnetic storm?. J. Geophys. Res..

[4] Denardini C.M., da Silva M.R., Gende M.A., Chen S.S., Fagundes P.R., Schuch N.J., Petry A., Resende L.C.A., Moro J., Padilha A.L. (2015). The South American K index: Initial steps from the Embrace magnetometer network. Braz. J. Geophys..

[5] Matzka J., Stolle C., Yamazaki Y., Bronkalla O., Morschhauser A. (2021). The Geomagnetic Kp Index and Derived Indices of Geomagnetic Activity. Space Weather.

[6] Campbell W.H. (2003). Introduction to Geomagnetic Fields.

[7] Johnston M.J.S. (1997). Review of electric and magnetic fields accompanying seismic and volcanic activity. Surv. Geophys..

[8] Integrated Plate Boundary Observatory Chile (IPOC). Magnetotellurics at IPOC. https://www.ipoc-network.org/observatory/magnetotellurics/magnetotellurics-at-ipoc/.

[9] Bartington Instruments. Shop. https://www.bartingtonshop.com/shop/.

[10] Boston College, Institute for Scientific Research South American Meridional B-Field Array (SAMBA). https://sites.bc.edu/magnetometers/.

[11] Love J.J., Chulliat A. (2013). An International Network of Magnetic Observatories. Eos Trans. Am. Geophys. Union.

[12] Denardini C.M., Chen S.S., Resende L.C.A., Moro J., Bilibio A.V., Fagundes P.R., Gende M.A., Cabrera M.A., Bolzan M.J.A., Padilha A.L. (2018). The Embrace Magnetometer Network for South America: Network description and its qualification. Radio Sci..

[13] Guizelli L.M., Denardini C.M., Moro J., Resende L.C.A., Chen S.S., Nyassor P.K. (2026). Assessing Magnetic Fluctuations Effects in the South American Sector in Relation to Global Variations Using Ksa Index, Kp Index, and Hybrid Formats During Quiet Times Across the Seasons of the 2020 Solar Minimum. J. Geophys. Res. Space Phys..

[14] Cordell D., Unsworth M.J., Díaz D. (2018). Imaging the Laguna del Maule Volcanic Field, central Chile using magnetotellurics: Evidence for crustal melt regions laterally-offset from surface vents and lava flows. Earth Planet. Sci. Lett..

[15] WDCKyoto World Data Center for Geomagnetism. https://wdc.kugi.kyoto-u.ac.jp/.

[16] EDA Instruments Inc (1984). Operation Manual FM-100B & FM-105B Three Component Fluxgate Magnetometers.

[17] FG Sensors (2022). FG-43 3-Axis Fluxgate Magnetometer System—Datasheet and User Manual.

[18] ARDC Amateur Radio Digital Communications Grant: Space Weather School Observatory for Chile: A Low-Cost Network to Study Space Weather from Schools on the Longest Country of the World. https://www.ardc.net/apply/grants/2024-grants/grant-space-weather-school-observatory-for-chile-a-low-cost-network-to-study-space-weather-from-schools-on-the-longest-country-of-the-world/.

[19] Leuzinger A., Taylor A. (2010). Magneto-Inductive Technology Overview.

[20] PNI Sensor Corporation (2013). RM3100 & RM2100 Geomagnetic Sensor: User Manual.

[21] Regoli L.H., Moldwin M.B., Pellioni M., Bronner B., Hite K., Sheinker A., Ponder B.M. (2018). Investigation of a low-cost magneto-inductive magnetometer for space science applications. Geosci. Instrum. Methods Data Syst..

[22] Regoli L., Moldwin M., Thoma J., Pellioni M., Bronner B. Four-Magnetometer Board for CubeSat Applications. Proceedings of the AIAA/USU Conference on Small Satellites.

[23] GNSS J.A.V.A.D. (2019). TRH-G2P OEM Board Datasheet. https://download.javad.com/sheets/TRH-G2P_OEM_Datasheet.pdf.

[24] Microchip Technology Inc (2020). MCP9808: +/− 0.5 °C Maximum Accuracy Digital Temperature Sensor.

[25] Hoffmann A.P., Moldwin M.B., Strabel B.P., Ojeda L.V. (2023). Enabling boomless CubeSat magnetic field measurements with the Quad-Mag magnetometer and an improved underdetermined blind source separation algorithm. J. Geophys. Res. Space Phys..

[26] Rabiu A.B., Mamukuyomi A.I., Joshua E.O. (2007). Variability of equatorial ionosphere inferred from geomagnetic field measurements. Bull. Astron. Soc. India.

[27] Chen S.S., Resende L.C.A., Denardini C.M., Chagas R.A.J., Da Silva L.A., Marchezi J.P., Moro J., Nogueira P.A.B., Santos A.M., Jauer P.R. (2023). The 14 December 2020 Total Solar Eclipse Effects on Geomagnetic Field Variations and Plasma Density Over South America. J. Geophys. Res. Space Phys..

[28] Gavazzi B., Alkhatib-Alkontar R., Munschy M., Colin F., Duvette C. (2017). On the Use of Fluxgate 3-Axis Magnetometers in Archaeology: Application with a Multi-sensor Device on the Site of Qasr ’Allam in the Western Desert of Egypt. Archaeol. Prospect..

[29] Strabel B.P., Regoli L., Moldwin M.B., Ojeda L., Shi Y., Thoma J.D., Narrett I.S., Bronner B., Pellioni M. (2022). Quad-Mag board for CubeSat applications. Geosci. Instrum. Methods Data Syst..

[30] Kim H., Witten D., Madey J., Frissell N.A., Gibbons J., Engelke W., Liddle A., Muscolino N., Visone J., Cao Z. (2024). Citizen science: Development of a low-cost magnetometer system for a coordinated space weather monitoring. HardwareX.

