# Supplementary material for: Comparative Performance Analysis of Five Magnetometers for Geomagnetic Monitoring
Source: Sensors (Basel). 2026 Jul 23;26(15):4700. doi: 10.3390/s26154700 (PMC13468621; doi:10.3390/s26154700)
Supplement: Supplementary file 1 [file sensors-26-04700-s001.zip › sensors-4350345-supplementary.pdf]

Supplementary Materials for

Comparative Performance Analysis of Five Magnetometers for Geomagnetic Monitoring

Guillermo Caro, Enrique Carrasco*, Joaquín Diaz Peña, Benjamín Urra, Enrique Rojo, Loreto Castro, Manuel Bravo, Victor Pinto, Marina Stepanova, Adán Godoy, Elías Ovalle, Clezio Marcos Denardini, Sony Su Chen

Correspondence to: enriquecarrasco@unach.cl

This PDF file includes

- Figures S1–S5

S1 Additional Figures

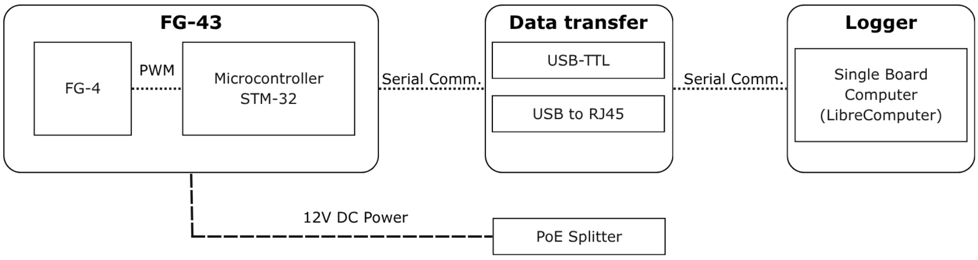

Figure S1: Wiring diagram of the FG-43 magnetometer.

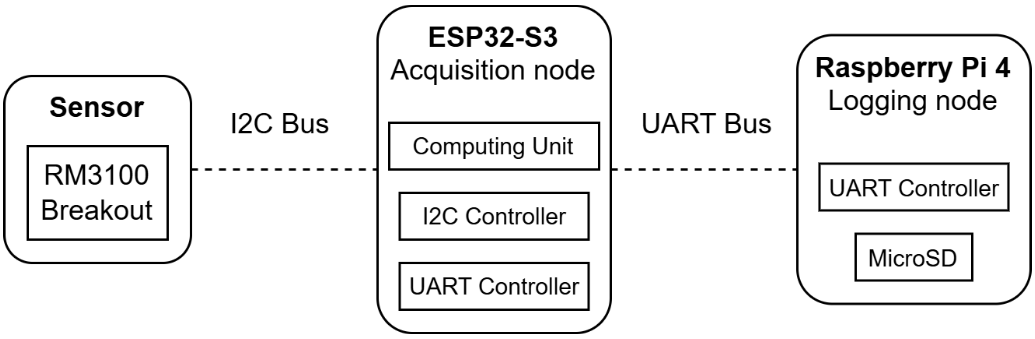

Figure S2: Wiring diagram of the RM3100 magnetometer.

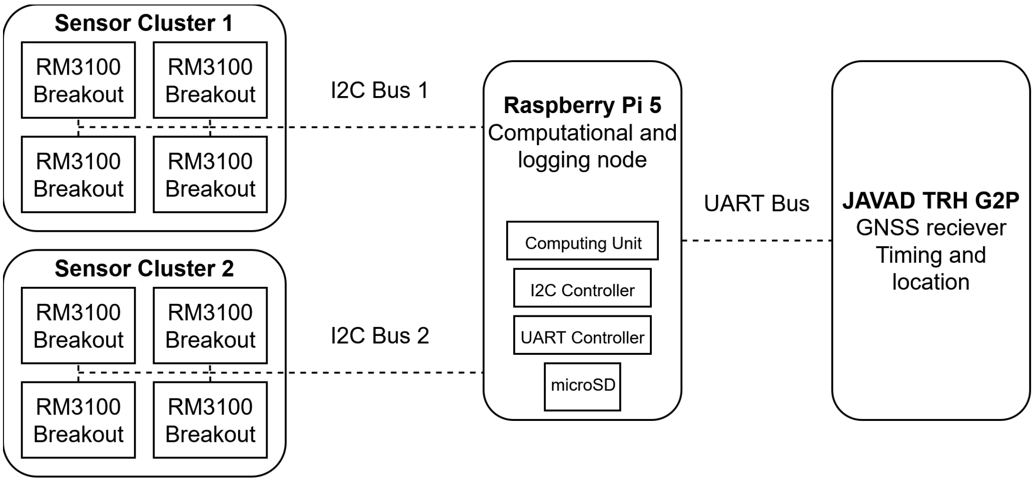

Figure S3: Wiring diagram of the RM3100 cluster.

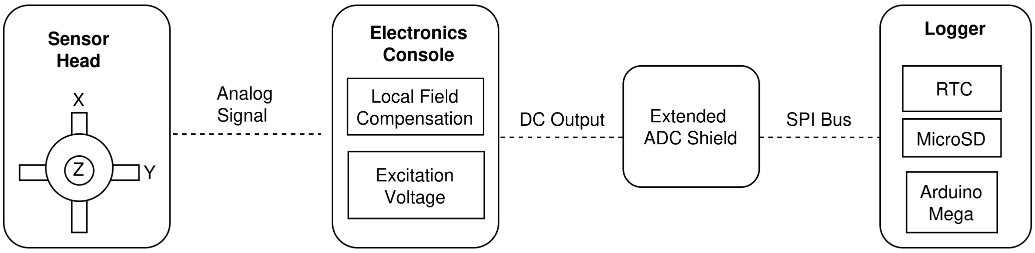

Figure S4: Wiring diagram of the FM-105B magnetometer.

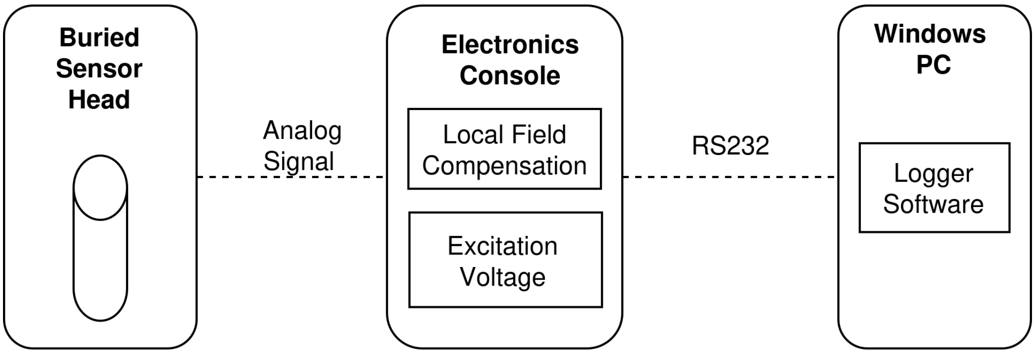

Figure S5: Wiring diagram of the EMBRACE magnetometer.
